# Mass Spectral Filtering by Mass-Remainder Analysis (MARA) at High Resolution and Its Application to Metabolite Profiling of Flavonoids

**DOI:** 10.3390/ijms22020864

**Published:** 2021-01-16

**Authors:** Tibor Nagy, Gergő Róth, Ákos Kuki, Miklós Zsuga, Sándor Kéki

**Affiliations:** 1 Department of Applied Chemistry, Faculty of Science and Technology, University of Debrecen, Egyetem tér 1, H-4032 Debrecen, Hungary; nagy.tibor@science.unideb.hu (T.N.); roth.gergo@science.unideb.hu (G.R.); kuki.akos@science.unideb.hu (Á.K.); zsuga.miklos@science.unideb.hu (M.Z.); 2 Doctoral School of Chemistry, University of Debrecen, Egyetem tér 1, H-4032 Debrecen, Hungary

**Keywords:** metabolomics, flavonoid, direct infusion mass spectrometry, mass spectral filtering, mass-remainder analysis, principal component analysis

## Abstract

Flavonoids represent an important class of secondary metabolites because of their potential health benefits and functions in plants. We propose a novel method for the comprehensive flavonoid filtering and screening based on direct infusion mass spectrometry (DIMS) analysis. The recently invented data mining procedure, the multi-step mass-remainder analysis (M-MARA) technique is applied for the effective mass spectral filtering of the peak rich spectra of natural herb extracts. In addition, our flavonoid-filtering algorithm facilitates the determination of the elemental composition. M-MARA flavonoid-filtering uses simple mathematical and logical operations and thus, it can easily be implemented in a regular spreadsheet software. A huge benefit of our method is the high speed and the low demand for computing power and memory that enables the real time application even for tandem mass spectrometric analysis. Our novel method was applied for the electrospray ionization (ESI) DIMS spectra of various herb extract, and the filtered mass spectral data were subjected to chemometrics analysis using principal component analysis (PCA).

## 1. Introduction

**Scheme 1:**
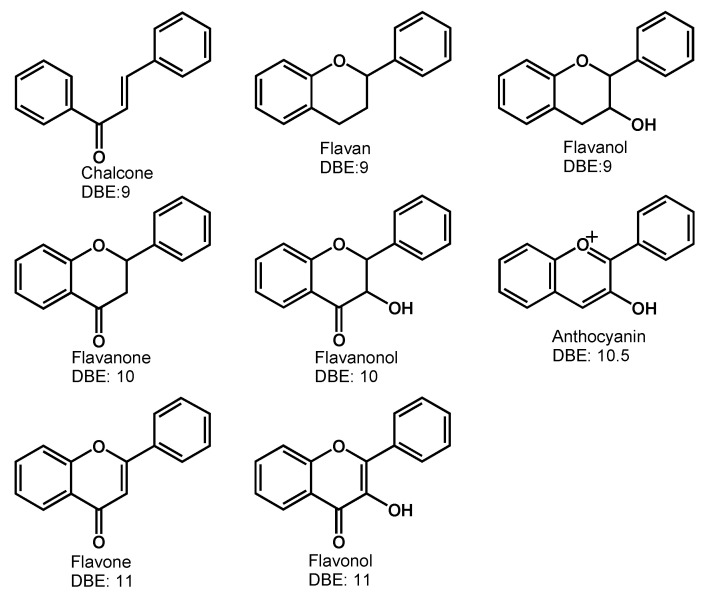
The structures of main flavonoid groups and their double bond equivalent (DBE) values.

Metabolomics is a comprehensive analysis that reveals the metabolome, i.e., all the metabolites of a biological system under study [1]. Metabolomics plays an increasingly important role in plant science [2]. In metabolic analysis different analytical approaches have been designed on several levels, such as “metabolite fingerprinting”, “metabolite profiling”, and “metabolite target analysis” [1,3]. Metabolic profiling focuses on the analysis of a group of metabolites or a class of compounds. An important class of plant metabolites is the flavonoids because of their potential health benefits and functions in plants [4]. Nearly 20,000 different flavonoids have now been characterized [5], with many of them showing interesting antioxidant, anti-inflammatory, anti-mutagenic and anti-carcinogenic properties [6,7]. They are active contributors to the health benefit of foods and beverages of plant origin, such as fruits, vegetables, tea, cocoa, and wine. Flavonoids have a C_6_-C_3_-C_6_ (A, C, B ring) core structure and depending on the position of the linkage between the C and B rings they can be divided into classes, while based on the degree of oxidation and saturation in the heterocyclic C-ring the flavonoids may be divided into several groups. For example, flavones, flavonols, flavanones, flavanonols, flavanols. In addition, the huge diversity of flavonoids arises from the number and complexity of substituents and presence of additional heterocyclic rings [6,7]. The structures of the main flavonoid groups are shown in Scheme 1.

The identification and quantification of flavonoids requires sophisticated, advanced analytical techniques. Mass spectrometry (MS) and nuclear magnetic resonance (NMR) spectroscopy, especially when they are coupled to chromatographic techniques, particularly liquid chromatography (LC), are the two leading analytical methods used for metabolic profiling of natural extracts. Significant advances in hyphenated techniques (LC–MS, LC–NMR), as well as in combined MS/NMR methods, have occurred over the last decades [3,8,9,10,11,12]. On the other hand, the mass accuracy and resolving power of high resolution mass spectrometry (HRMS) has dramatically improved the detection and identification of compounds in complex natural samples and, therefore, has increased the relevance of non-hyphenated MS methods. Direct infusion mass spectrometry (DIMS) can significantly increase the analytical throughput compared to LC–MS [3,13]. However, the rapid identification of hundreds or thousands of mass peaks arising in direct injection of complex natural samples requires advanced data mining techniques. Mass defect filtering (MDF) of complex MS data has been used for selectively detecting compound classes having similar mass defects (where mass defect is the difference between a compound’s exact mass and its nominal mass). The MDF process can facilitate the detection of the target compounds by the selective removal of all ions that fall outside of the preset filter mass range window [14,15,16,17]. However, if the aim is the comprehensive analysis of the flavonoids in complex natural samples, a wide mass defect window should be applied that would likely include many false peaks. The flavonoids fall into the mass defect segment where numerous possible chemical compositions may be valid [18].

Besides the MDF process, the Kendrick Mass Defect (KMD) [19] and related methods [20,21,22] (Re-KMD) are applied for the analysis of complex mass spectra, however, it is not widespread in metabolomics.

Recently, we have developed a data mining algorithm, called mass-remainder analysis (MARA) and its improved variant, multi-step mass-remainder analysis (M-MARA) for the processing and assessment of complex mass spectra [23,24,25,26,27]. In this paper, we propose a novel method for the comprehensive filtering and screening of flavonoids in the DIMS spectra of natural extracts.

## 2. Results and Discussion

**Figure 1:**
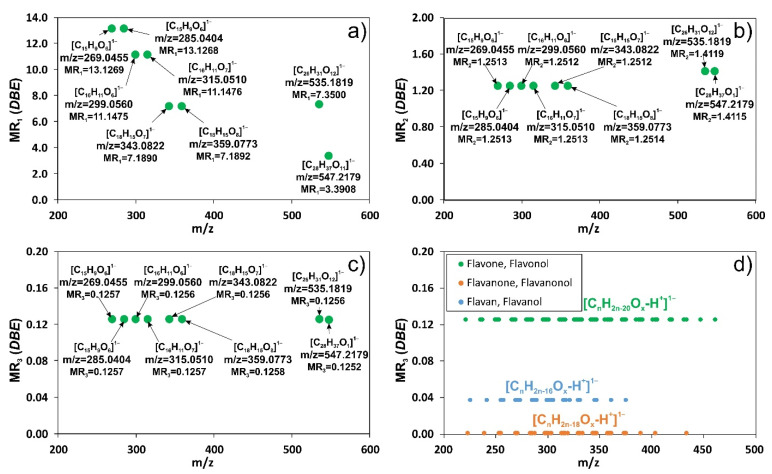
(**a**) MR_1_ (*DBE*) versus *m/z* plot for eight flavonoids detected in the extract of the medical plant yarrow (*Achillea millefolium* L.). (**b**) MR_2_ (*DBE*) *vers*us *m/z* plot for eight flavonoids detected in the extract of the medical plant yarrow (*Achillea millefolium* L.). (**c**) MR_3_ (*DBE*) versus *m/z* plot for eight flavonoids detected in the extract of the medical plant yarrow (*Achillea millefolium* L.). (**d**) MR_3_ (*DBE*) versus *m/z* plot calculated from the theoretical *m/z* values of about one hundred flavonoids. (*DBE* in parenthesis indicates that the double bond equivalent values were revealed as the characteristic difference.).

As it was detailed in our previous works [23,24,25,26], mass-remainder analysis (MARA) and its multistep extension (M-MARA) are able to produce simplified graphical representations of the mass spectra, facilitating thereby the recognition, filtering, and grouping of the detected compounds. M-MARA simply performs sequential modulo operations on the measured *m/z* values in order to eliminate the homologous connections present in a complex system. Equation (1) reveals the general formula for the three-step M-MARA [26]:

MR_3_ = [(*m/z* MOD R_1_) MOD R_2_] MOD R_3_ (1)

where the modulo (MOD) operation finds the mass remainder (MR_n_) after the division by R_n_ (*n* = 1, 2, 3). It can easily be seen, that MARA is highly effective for data mining of mass spectra containing numerous peaks with periodicities generated by e.g., a repeat unit (polymers) or the CH_2_ group (hydrocarbons). Therefore, at a first glance, the application of MARA might seem to be challenging and questionable for processing the mass spectra of complex natural extracts composed of very diverse flavonoid constituents. However, by careful selection of the M-MARA base units (R_n_ in Equation (1)), most of the differences in the elemental composition of the flavonoids can be eliminated, and a single characteristic difference can be obtained and used further for grouping and effective filtering of the constituents. An interesting and promising approach is to sort the flavonoids according to their double bond equivalent (DBE) values. The main steps of our analysis are summarized and an example is detailed later. In the first step, the exact mass of the oxygen atom was chosen as the divisor of the modulo operation (R_1_ = 15.994915). As a simplified example, Figure 1a shows the MR_1_ versus *m/z* plot of eight flavonoids detected in the extract of the medicinal plant yarrow (*Achillea millefolium* L.).

**Figure 2:**
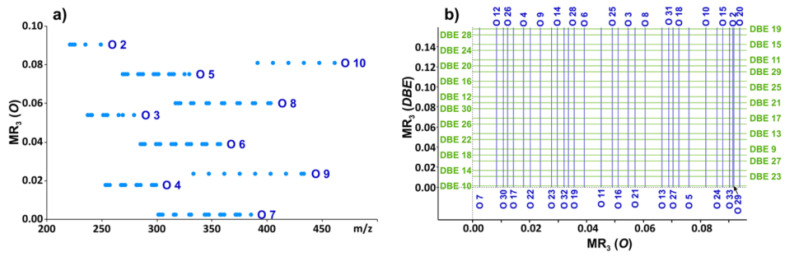
(**a**) MR_3_ versu*s m/z* plot calculated from the theoretical *m/z* values of about one hundred flavonoids (applied for Figure 1d). (*O* in parenthesis indicates that the number of oxygen atoms were revealed as the characteristic difference.) (**b**) MR_3_(*DBE*)—MR_3_(*O*) mesh used as a mass spectral filter.

As it can be seen in Figure 1a, compounds differing only by the number of oxygen atoms have the same MR_1_ values and are aligned in a line. Moreover, Figure 1a also reveals another homologous connection that can be eliminated in the second MARA step. A characteristic difference of MR_1_ = 1.97927 appears between the neighboring lines (dots) that corresponds to the mass difference between oxygen and CH_2_ group. Using R_2_ = 1.97927 in the next step, MARA assigns the same MR_2_ value to most of the compounds differing only by the number of oxygen atoms and CH_2_ groups (see Figure 1b). Using the MR_2_ difference in Figure 1b as the base unit of the third MARA step (R_3_ = 0.160795, i.e., corresponding to the mass of 8CH_2_-7O), the *m/z* range of the filtering can be extended, and thereby the MR_3_ versus *m/z* plot (Figure 1c) shows merely a single line. Figure 1d shows the MR_3_ versus *m/z* plot including the theoretical *m/z* values of more than one hundred flavonoids. Accordingly, three groups were distinguished based on the DBE values, flavans plus flavanols, flavanones plus flavanonols, and flavones plus flavonols, having the DBE values 9, 10, and 11, respectively. However, if the goal is a comprehensive mass spectral filtering of the flavonoid compounds, a wider DBE range should be considered. Due to the large number and complexity of substituents and presence of additional heterocyclic rings, the flavonoid species cover a DBE range of 9–30 [28,29]. (See Figure 2, which will be discussed later). By using this DBE range, our MARA filtering method removes the interference mass spectral peaks, which do not correspond to the elemental composition C_x_H_2x+2-2*DBE_O_y_, where DBE = 9–30, x and y are arbitrary integers. Now we will use the acronym M-MARA(*DBE*) for this first M-MARA run, which utilizes the DBE value as the characteristic difference for grouping and filtering of the flavonoid compounds. In order to decrease the number of false peaks remaining in the mass spectrum after filtering, a second M-MARA process using a different base unit set (R_1_, R_2_, and R_3_) was performed. M-MARA(*O*) calculates MR_3_ values, which are independent from the elemental composition, except for the number of oxygen atoms in the flavonoid constituents. The divisors of M-MARA(*O*) are R_1_ = 14.01565, R_2_ = 2.01565, and R_3_ = 0.0939 corresponding to the mass of CH_2_, H_2_ and 7H_2_-CH_2_, respectively. Figure 2a shows the output of M-MARA(*O*) algorithm, applying the same *m/z* values (about one hundred flavonoids) as M-MARA(*DBE*) (see Figure 1d). 

Although the grouping of compounds does not correspond to the usual classification of the flavonoids (e.g., flavones, flavonols, flavans, flavanols, etc.), the MR_3_ values of M-MARA(*O*) can be used for refining the filtering algorithm and for assigning the number of oxygens in a detected compound. By rotating the MR_3_ plot of M-MARA(*O*) and combined with that of M-MARA(*DBE*), an overlay map can be constructed (see Figure 2b). Figure 2b illustrates our filtering algorithm: if the [MR_3_(*O*); MR_3_(*DBE*)] point, calculated from the *m/z* value of a mass spectral peak, does not coincides with an intersection of Figure 2b, this mass peak is filtered out. Here, we would like to emphasize again that the calculation of the MR_3_ values requires simple divisions. The lower and upper DBE and O values of the filter (shown in Figure 2b) were determined based on the flavonoid database [28,29]. The smallest differences between the MR_3_(O) and MR_3_(DBE) values are 0.00113 and 0.00588, respectively. Our novel M-MARA method can be used if the applied instrument is capable of distinguishing these differences. The effect of accuracy on the efficiency of elemental composition determination was discussed by Kim et al. [18].

The correlation between the DBE value and the number of oxygen atoms of the flavonoids offers an additional filtering criterion. 

**Figure 3:**
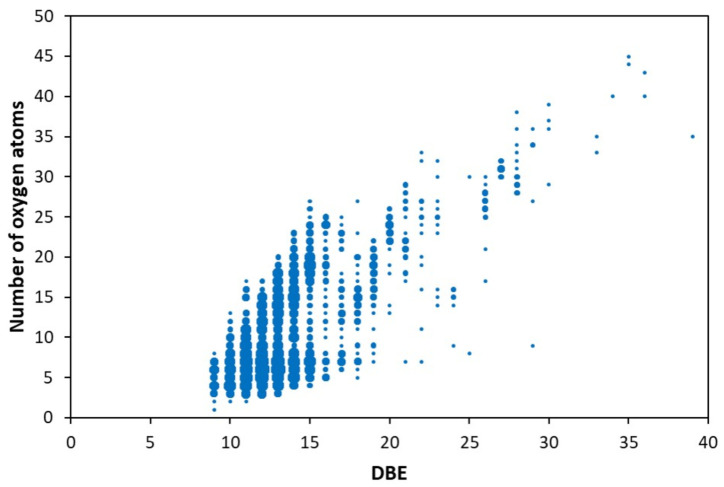
The DBE values and the number of oxygen atoms of the compounds in the flavonoid database [28,29]. The Pearson’s correlation coefficient r = 0.76.

As Figure 3 shows, higher DBE values imply higher number of oxygen atoms (the Pearson’s correlation coefficient r = 0.76), decreasing thus the number of valid intersections in Figure 2b, e.g., the number of [MR_3_(*O*); MR_3_(*DBE*)] points of the mass peak filter. Furthermore, there are also strong positive correlations between the DBE versus *m/z* (r = 0.85) and the number of oxygen atoms versus *m/z* values (r = 0.97) (see Figures S1 and S2). For example, at *m/z* 600, the DBE value and the number of oxygen atoms of the flavonoid compounds are delimited between 11–19 and 8–17, respectively. 

**Figure 4:**
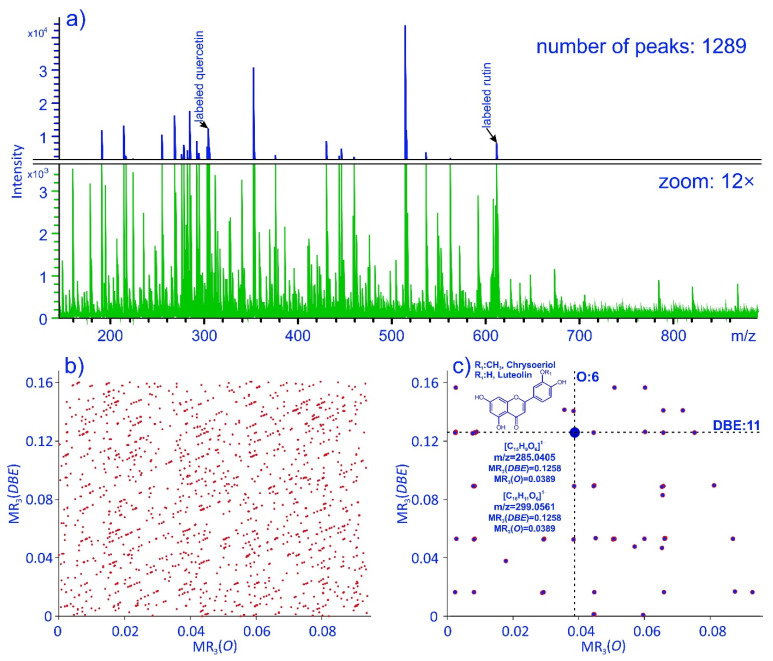
(**a**) ESI-DIMS spectrum of yarrow (*Achillea millefolium* L.) extract. (**b**) MR_3_(*O*)—MR_3_(*DBE*) plot of a yarrow (*Achillea millefolium* L.) extract. (**c**) Filtered MR_3_(*O*)—MR_3_(*DBE*) plot of a yarrow extract.

In order to demonstrate the usefulness and capability of our novel mass filtering method, we applied it for screening of flavonoids in various herb plant extracts. As a representative example, Figure 4b shows the MR_3_(*O*)—MR_3_(*DBE*) plot constructed from the ESI-DIMS mass spectrum (shown in Figure 4a) of a yarrow (*Achillea millefolium* L.) extract (sample 1).

Each point in Figure 4b corresponds to one or more peaks of the mass spectrum presented in Figure 4a. By applying our MR_3_(*O*)—MR_3_(*DBE*) filter (see Figure 2b) algorithm, the interference mass spectral peaks can be removed, and therefore, Figure 4c reveals only the points, which correspond to possible flavonoid constituents. For example, the mass peaks at *m/z* 285.0405 and 299.0562 belong to the [0.0389; 0.1258] point in the [MR_3_(*O*); MR_3_(*DBE*)] plane, corresponding to six oxygens in the molecule and DBE = 11 (see Figure 4c). The exact masses enable the assignment of the elemental compositions, such as [C_15_H_9_O_6_]^1−^ (*m/z* 285.0405) and [C_16_H_11_O_6_]^1−^ (*m/z* 299.0562), and suggest a possible identification as luteolin and chrysoeriol (a methoxy derivative of luteolin), respectively [30]. 

**Scheme 2:**
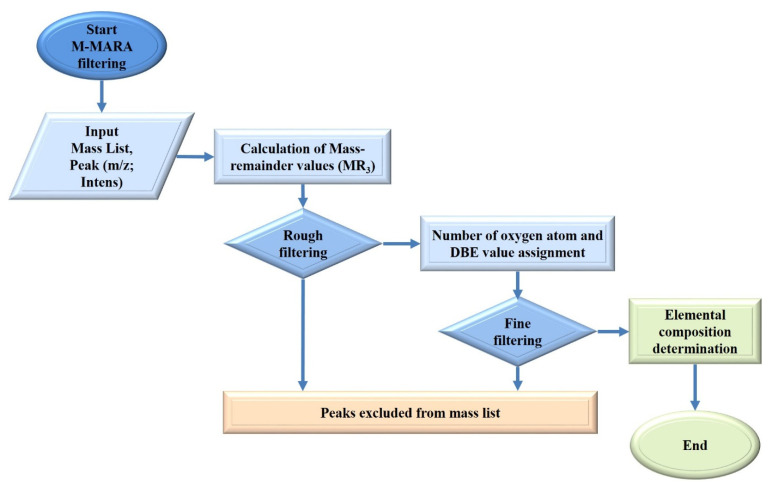
Flowchart of the Multistep Mass Remainder Analysis (M-MARA) flavonoid filtering algorithm.

To summarize, the steps of our M-MARA flavonoid filtering algorithm are the following (see Scheme 2):

(1) Calculation of the *Mass-remainder* values by Equation (1). MR_3_(*O*) and MR_3_(*DBE*) are calculated for all of the observed mass peaks (above an intensity threshold) of the ESI-DIMS mass spectrum. The divider base units (R_1_, R_2_, R_3_ in Equation (1)) are for M-MARA(*O*): 14.01565, 2.01565, and 0.0939, and for M-MARA(*DBE*): 15.994915, 1.97927, and 0.160795.
(2) Rough filtering. The potential flavonoid MR_3_(*O*) and MR_3_(*DBE*) values are compiled in the MR-Table (see Figure 2b, Supplementary Table S1). A mass peak is removed, if its calculated MR_3_ values have not been found in the table.
(3) Number of oxygen atom assignment and DBE value assignment. There is one-to-one correspondence between MR_3_(*O*) and the number of oxygen atoms in the molecule, and between MR_3_(*DBE*) and DBE value of the compound. The MR-Table (Table S1) can easily be used for these assignments.
(4) Fine filtering. The correlations between the DBE versus *m/z* and number of oxygen atoms versus *m/z* values (see Figures S1 and S2) offer additional filtering criteria. Based on the flavonoid database [28,29], an *m/z* range was determined for every number of oxygen atoms and DBE value. If the *m/z* value of a mass peak does not fall into the ranges corresponding to its O and DBE values, the peak is eliminated.
(5) Elemental composition determination. Once the number of oxygen atoms (O) and the DBE values have been assigned, the general formula of the compound follows as C_x_H_2x+2-2*DBE_O_O_, where *x* is the only unknown variable. Knowing the exact *m/z* of the deprotonated [M-H^+^]^–^ ion, *x* can easily be calculated by Equation (2):
  *x* = (*m/z* + 1.007276 − *O* × 15.994915 + 2 × *DBE* × 1.007825 − 2 × 1.007825)/14.01565 (2)
  where 1.007825, 1.007276, 15.994915, and 14.01565 are the exact masses of H, H^+^, oxygen and CH_2_, respectively.

In the following, we will show, through a simple example, how our algorithm works. Let the *m/z* value of the compound in question be 285.0405. The steps of our M-MARA flavonoid-filtering approach are as follows:

(1) MR_3_(*O*) = 0.0389, MR_3_(*DBE*) = 0.1258
(2) The search in the MR-Table have found the calculated MR_3_ values, the compound is a potential flavonoid.
(3) The number of oxygen atom and DBE values are O = 6, DBE = 11.
(4) The *m/z* ranges corresponding to O = 6, DBE = 11 are [283.0236; 557.2921] and [221.0596; 655.2256], respectively. The measured *m/z* = 285.0405 falls into these intervals, the compound is accepted as a flavonoid.
(5) Equation (2) gives *x* = 15, so the elemental composition of the compound is C_15_H_10_O_6_ (see Figure 4c)

As the detailed steps and the example above demonstrate, our M-MARA flavonoid-filtering does not require any sophisticated, advanced mathematical and software tools, it needs simple mathematics, table searches and *IF* conditions. These operations can easily be implemented in a regular spreadsheet software.

The suggested application area of our M-MARA filtering method is the comprehensive metabolite profiling of flavonoids in herb extract, foods and beverages of plant origin. As a pilot study to test our approach, the flavonoid analysis of altogether 15 commercially available brands of three herbs, such as yarrow (*Achillea millefolium* L.), elder (*Sambucus nigra* L.), and birch (*Betula* L.) were performed. Table 1 summarizes the very basic statistics related to the number of mass peaks. 

The last row in Table 1 reports the number of mass peaks identified by the time-consuming search in the flavonoid database [28,29]. For example, processing the ESI-DIMS spectra of Sample No. 1 (yarrow extract), M-MARA filtered 66 peaks as potential flavonoids, while the database search, based on the exact mass, found 51 peaks. It does not mean, however, that all the 66 − 51 = 15 peaks are false hits, these peaks may include, perhaps novel, flavonoids or flavonoid derivatives. Furthermore, the mass peaks found by M-MARA and not included in the flavonoid database typically have very low peak intensities, as seen in Figure 4a.

Furthermore, once the mass peaks of the flavonoids are selected by M-MARA-filtering, multivariate analysis such as principal component analysis (PCA) can serve as an efficient tool for discrimination of the type or brand of natural origin samples based on their flavonoid contents.

The PCA highlights the differences among the samples creating principal components. Applying the intensities only do not reflect the concentration ratios of the flavonoids and flavonoid glycosides, due to the different ionization efficiencies and/or matrix effects. In order to overcome such cumbersome, isotope dilution [31], with deuterium labeled rutin and quercetin as internal standard (IS), was applied and all the measured intensities were corrected based on the labeled IS peaks. The ratio of the ionization efficiencies was around 2:1 quercetin to rutin.

**Figure 5:**
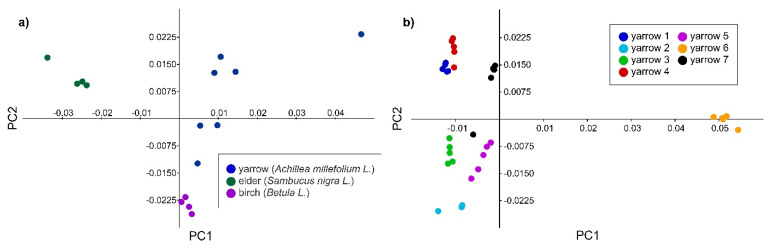
Principal Component Analysis (PCA) score plots for the filtered ESI-DIMS mass spectra of (**a**) the different herbs (variance % PC1: 43.4%, PC2: 31.5%), (**b**) different brands of yarrow (variance % PC1: 66.0%, PC2: 32.0%).

Figure 5a demonstrates the two-dimensional PCA score scatter plot for the ESI-DIMS mass spectra, after filtered by M-MARA, of the fifteen herb extract (Sample No 1–No. 15). As seen in Figure 5a, significant clustering can be observed based on the herb type. In order to examine whether PCA is able to discriminate between the different brands of the same herb plant, each of the seven yarrow samples was measured five times. As it can be seen in Figure 5b, that PCA grouped the yarrow samples according their origin. 

## 3. Materials and Methods

### 3.1. Chemicals

Ethanol 96% (puriss.) was purchased from Molar Chemicals (Halásztelek, Hungary). Quercetin dihydrate 97%, ACN (acetonitrile) and deuterium oxide were received from VWR International (Leuven, Belgium). Sodium hydroxide (puriss.) was obtained from Sigma Aldrich (Taufkirchen, Germany). Rutin was isolated from a Japanese pagoda tree (*Sophora japonica*) through an alcohol extraction. Water was purified by a Direct-Q water system (Millipore, Molsheim, France).

### 3.2. Synthesis of Deuterium Labeled Rutin and Quercetin

The synthesis of the labeled rutin and quercetin was done based on the method described by Hiraoka et al. [32]. Rutin (400 mg) and quercetin (62 mg) were weighed into a round-bottomed flask. The compounds were dissolved in 4.0 and 12.6 g D_2_O containing 10 mg/g NaOH. The solutions were heated up to 95 °C and stirred with a magnetic stirrer for 8 h under argon atmosphere. In the next step, the reaction mixtures were freeze-dried. The obtained powders were dissolved again in 4.0 and 12.6 g D_2_O and additional heating (95 °C) was applied for 8 h, stirred with a magnetic stirrer. After cooling down, the reaction mixture was treated by 13.3 and 20.7 mL acetic acid solution (10 *v/v* %) and stored at 4 °C overnight. The precipitate was filtered and washed with cold water and dried under vacuum. The HPLC-UV chromatograms and the mass spectra of the deuterated samples can be found in the Supporting Information as Figures S4 and S5.

### 3.3. Extraction of Samples

15 commercially available brands of three herbs, such as yarrow (*Achillea millefolium* L.), elder (*Sambucus nigra* L.), and birch (*Betula* L.) were investigated. The manufacturer and the location of collections are in the Supporting Information as Table S2. Plant samples were ground, after which the powders were swollen in ethanol: water-7:3 (*v/v*) for 16 h. After the swelling process, the diluted samples were sonicated for an hour and then filtered. The filtrates were dried under vacuum to constant weights.

### 3.4. Electrospray Quadrupole Time-of-Flight Mass Spectrometry (ESI-QTOF MS)

The high resolution mass spectrometric measurements were performed using a Maxis II type Qq-TOF MS instrument (Bruker Daltonics, Bremen, Germany) equipped with an electrospray ion-source where the spray voltage was 3.5 kV. N_2_ was applied as drying (200 °C, 4.0 L/min) and nebulizer gas (0.5 bar). The mass spectra were recorded by means of a digitizer at a sampling rate of 2 GHz. The mass accuracy of the instrument is <600 ppb (internal calibration), the resolution is 40,000 at *m/z* 400 (FWHM). Each spectrum was calibrated internally with the sodium formate clusters. The spectra were evaluated with the Compass DataAnalysis 4.4 software (Bruker Daltonics, Bremen, Germany). The sample solutions were injected by a 7100 model capillary electrophoresis instrument without any separation. A fused silica capillary (70 cm × 7.5 µm (id)) was applied, with 130 s, 3 bar preconditioning, 40 s, 2 bar injection and 100 s, 3 bar as postconditioning. The samples were dissolved in ethanol: water 1:1 (*v/v*) with 0.02% formic acid at a concentration of 60 µg/mL on a dry basis. The concentrations of the deuterated internal standards were 0.146 and 0.210 µg/mL quercetin and rutin, respectively.

### 3.5. Liquid Chromatography (HPLC-UV-MS)

The characterization of the deuterated standard materials was performed by liquid chromatograph—mass spectrometry measurements. For chromatography, a Waters 2695 Separations Module (Waters, Milford, MA, USA) equipped with a thermostable autosampler (5 °C) and a column module (40 °C), a Waters 2996 photodiode-array detector and a VDSphere PUR 100 C18-M-SE (4.6 × 150 cm, 5 µm) column. Gradient elution was used with 1.5 mL/min flow rate. Mobile phase A was ACN (acetonitrile) containing 0.02% formic acid and mobile phase B was water containing 0.02% formic acid. The gradient condition was as follows: initially 20% A and 80% B, 0–20 min linear change to 50% A and 50% B, 20–30 min linear change to 90% A and 10% B and held this setting for additional 10 min. The analytes were detected at λ = 240 nm. Mass spectrometric detection was carried out by a MicroTOF-Q type Qq-TOF MS instrument (Bruker Daltoniks) using an ESI-source with negative ion mode. The spray voltage was set to 4.5 kV. The nebulizer pressure was 1.6 bar, while, the temperature of the drying gas (N_2_, 7 L/min) was kept at 200 °C. The data evaluation was performed by the DataAnalysis 3.4 software from Bruker.

## 4. Conclusions

Multi-step Mass-remainder analysis (M-MARA) was successfully applied for data mining of complex, peak-rich ESI-DIMS mass spectra of various herb extracts. M-MARA eliminated most of the differences in the elemental composition of the flavonoids, the target class of our study, and a single characteristic difference was highlighted and used for filtering. Moreover, our flavonoid-filtering algorithm performs the mass remainder calculation twice, using different divisors, decreasing thereby the number of false hits of the mass filter, and facilitating the determination of the elemental composition. A huge benefit of our method is the high speed and the low demand for computing power and memory that enables the real time application. For example, the *Auto MSMS* feature of the mass spectrometer’s acquisition software can be extended by a filtering script or module, and applied for the automatic recognition, isolation and fragmentation of the flavonoids. In addition to the elemental composition, tandem mass spectra enable the distinction and identification of the flavonoid isomers as well.

## Figures and Tables

**Table 1:** The number of *m/z* peaks in the ESI-DIMS spectra of herb extracts before and after M-MARA filtering, and identified by database search.

Type		Yarrow (*Achillea* *millefolum* L.)							Elder (*Sambucus nigra* L.)				Birch (*Betula* L.)			
No.		1	2	3	4	5	6	7	8	9	10	11	12	13	14	15
No. of peaks	in the spectra	1258	889	883	1254	842	1070	1516	1251	1293	1067	1033	1578	1180	1662	1590
	M-MARA	66	15	52	65	53	58	56	49	35	52	40	60	57	67	63
	database search	51	9	36	45	40	45	43	37	28	41	31	41	45	45	46

## Data Availability

Data is contained within the article or supplementary material.

## Supplementary Materials

The following are available online at https://www.mdpi.com/1422-0067/22/2/864/s1.
