# Supplementary material for: Mass Spectral Filtering by Mass-Remainder Analysis (MARA) at High Resolution and Its Application to Metabolite Profiling of Flavonoids
Source: Int J Mol Sci. 2021 Jan 16;22(2):864. doi: 10.3390/ijms22020864 (PMC7830504; doi:10.3390/ijms22020864)
Supplement: Supplementary file 1 [file ijms-22-00864-s001.pdf]

Supporting Information

Mass spectral filtering by Mass-Remainder Analysis (MARA) and its application to metabolite profiling of flavonoids

Tibor Nagy[1], Gergő Róth[1,2], Ákos Kuki[1], Miklós Zsuga[1], Sándor Kéki[1]

[1]*Department of Applied Chemistry, Faculty of Science and Technology, University of Debrecen, Egyetem tér 1, H-4032 Debrecen, Hungary*

[2]*Doctoral School of Chemistry, University of Debrecen, Egyetem tér 1, H-4032 Debrecen, Hungary*

Table of Content

TABLE S1 MR_3 VALUES, NUMBER OF O AND NUMBER OF DBE WITH MINIMUM AND MAXIMUM M/Z LIMITS 2

TABLE S2. THE ANALYZED SAMPLES, THEIR BRANDS AND PLACE OF COLLECTION.......... 3

FIGURE S1. DBE VERSUS M/Z PLOT OF THE COMPOUNDS IN THE FLAVONOID DATABASE [1,2]. THE PEARSON'S CORRELATION COEFFICIENT R = 0.85 4

FIGURE S2. NUMBER OF OXYGEN ATOMS VERSUS M/Z PLOT OF THE COMPOUNDS IN THE FLAVONOID DATABASE [1,2]. THE PEARSON'S CORRELATION COEFFICIENT R = 0.97. 4

FIGURE S3. NORMALIZED INTENSITY VERSUS M/Z OF THE MASS PEAKS FILTERED BY M-MARA PROCESSING THE ESI-DIMS SPECTRA OF SAMPLE NO. 1 (YARROW EXTRACT).......... 5

FIGURE S4. THE HPLC-UV CHROMATOGRAMS OF TH EDEUTERIUM LABELED RUTIN AND QUERCETIN 6

FIGURE S5. THE MASS SPECTRA OF THE DEUTERIUM LABELED RUTIN AND QUERCETIN AND THE FITTING OF THE SIMULATED ISOTOPE PATTERNS OF COMPOUNDS WITH DIFFERENT DEUTERIUM CONTENT. 6

Table S1 MR_3 values, number of O and number of DBE with minimum and maximum m/z limits

$MR_3(O)$	Number of *O*	m/z_{min}	m/z_{max}	$MR_3(DBE)$	*DBE*	m/z_{min}	m/z_{max}
0.0905	2	221.0596	255.023	0.0377	9	209.096	361.1305
0.0541	3	237.0545	387.1978	0.0013	10	237.0909	553.3547
0.0177	4	241.0494	529.3335	0.1258	11	221.0596	655.2256
0.0752	5	267.0287	543.3128	0.0894	12	263.0702	697.2361
0.0389	6	283.0236	557.2921	0.0530	13	261.0545	771.2365
0.0025	7	299.0185	589.2244	0.0166	14	305.0443	831.2213
0.0600	8	317.0291	695.2662	0.1410	15	319.0236	963.2635
0.0236	9	333.024	801.3081	0.1046	16	357.0756	985.3206
0.0811	10	380.991	549.1778	0.0682	17	399.0862	921.3046
0.0447	11	396.9859	693.2353	0.0319	18	417.024	971.2322
0.0084	12	444.9529	605.1677	0.1563	19	537.1907	875.2264
0.0659	13	460.9478	659.1782	0.1199	20	659.1758	1021.284
0.0295	14	490.9584	725.1888	0.0835	21	589.222	1109.3
0.0870	15	524.9097	753.1837	0.0471	22	573.1907	1227.327
0.0506	16	540.9046	773.2099	0.0107	23	713.15	1181.285
0.0142	17	573.018	823.1892	0.1351	24	685.2431	769.1786
0.0717	18	620.985	801.2623	0.0987	25	695.2638	1173.295
0.0354	19	620.8614	821.2886	0.0624	26	823.1868	1199.311
0.0929	20	669.0933	887.2991	0.0260	27	1225.324	1285.348
0.0565	21	741.1508	891.294	0.1504	28	1149.272	1421.348
0.0201	22	730.9524	921.3046	0.1140	29	801.3057	1485.453
0.0776	23	797.1406	963.3152	0.0776	30	1091.157	1489.375
0.0412	24	813.1355	983.3414				
0.0048	25	857.1617	1077.289				
0.0624	26	933.2506	1107.3				
0.0260	27	963.2611	1123.295				
0.0835	28	1079.287	1163.29				
0.0471	29	1091.157	1193.3				
0.0107	30	1135.277	1253.358				
0.0682	31	1225.288	1283.368				
0.0319	32	1181.283	1285.348				
0.0894	33	1227.325	1387.358				

Table S2. The analyzed samples, their brands and place of collection

Sample number	Sample name	Manufacturer	Location
1	yarrow (floral sprout), Millefolii herba	Herbária patikája	Székkutas (Hungary)
2	yarrow, Millefolii herba	JuvaPharma	Felsőpakony (Hungary)
3	yarrow, Achillea herba	adamo-fitt	Fót (Hungary)
4	yarrow, Millefolii herba	Mama gyógynövényei	Isaszeg (Hungary)
5	yarrow, Millefolii herba	Gyógyfű Tea	Sóskút (Hungary)
6	yarrow, Ha. Millefolii	Boszy Teák	Sóskút (Hungary)
7	yarrow (floral sprout), Millefolii herba	Herbária patikája	Székkutas (Hungary)
8	goldenrod, Solidaginis Herba	Gyógyfű Tea	Sóskút (Hungary)
9	goldenrod, Solidaginis Herba	Mama gyógynövényei	Isaszeg (Hungary)
10	goldenrod, Solidaginis Herba	Mecsek Tea	Pécsvárad (Hungary)
11	goldenrod, Virgae aurea herba	adamo-fitt	Fót (Hungary)
12	elderflower, Sambuci flos	adamo-fitt	Fót (Hungary)
13	elderflower, Sambucii flos	JuvaPharma	Felsőpakony (Hungary)
14	elderflower, Sambuci flos	Mama gyógynövényei	Isaszeg (Hungary)
15	elderflower, Sambuci flos	natúr TEA	Pap (Hungary)
16	birch leaves, Betulae folium	Mecsek Tea	Pécsvárad (Hungary)
17	birch leaves, Betulae folium	Gyógyfű Tea	Sóskút (Hungary)
18	birch leaves, Betulae folium	Mama gyógynövényei	Isaszeg (Hungary)
19	birch leaves, Betulae folium	Herbária patikája	Székkutas (Hungary)

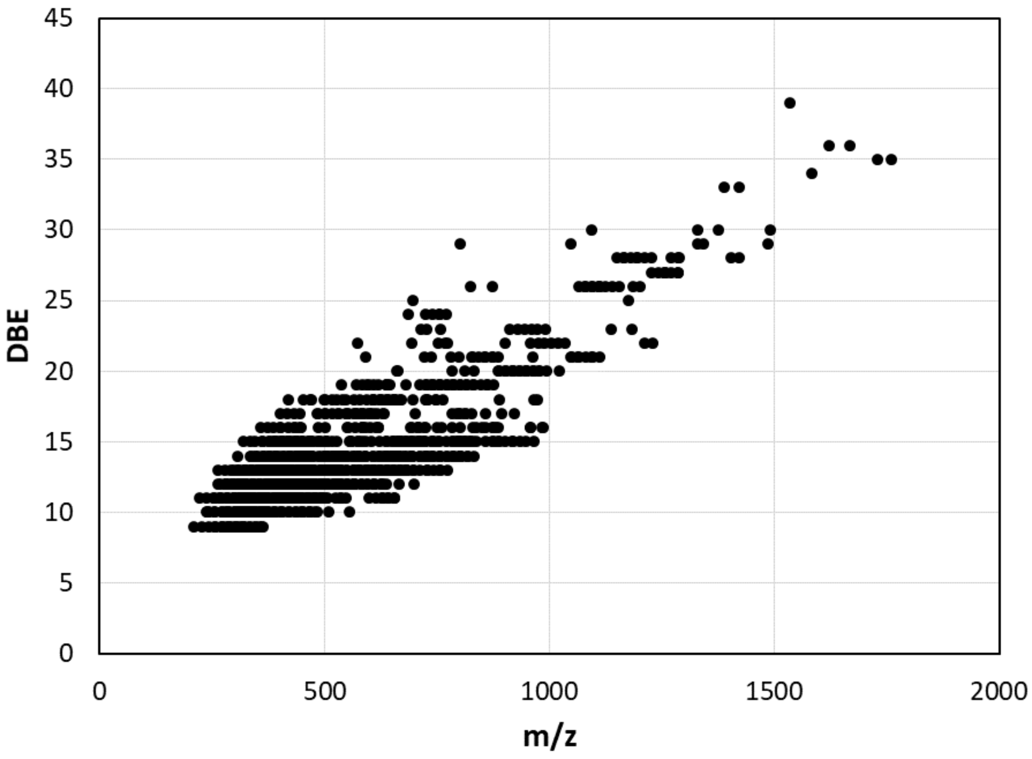

Figure S1. DBE versus m/z plot of the compounds in the flavonoid database [1,2]. The Pearson's correlation coefficient r = 0.85

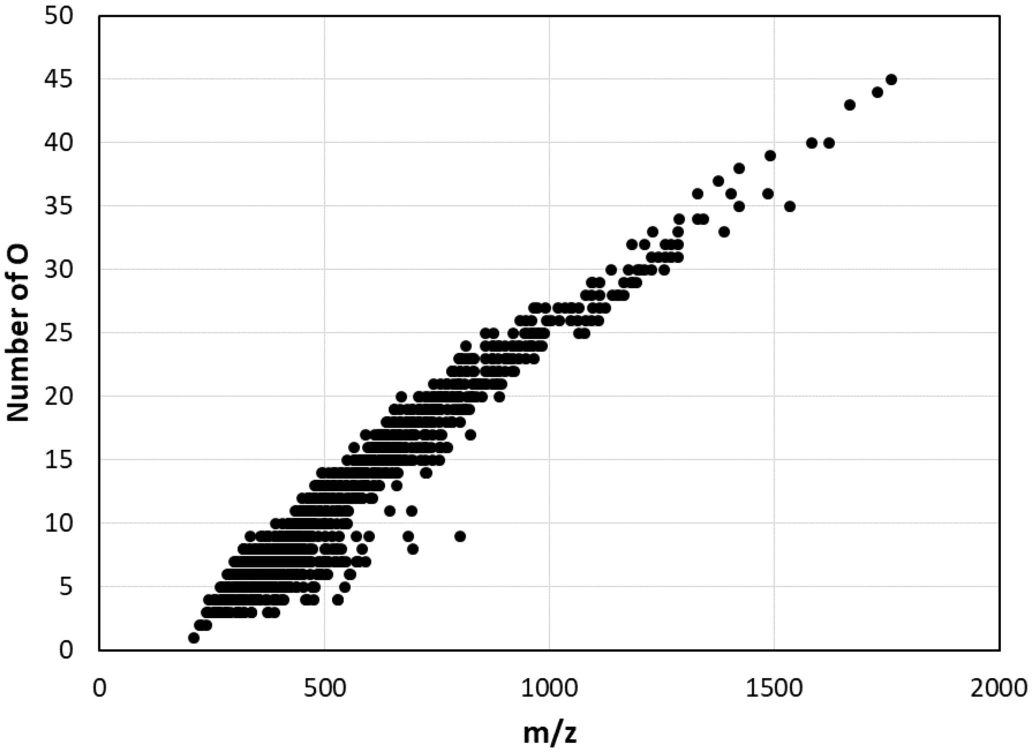

Figure S2. Number of oxygen atoms versus m/z plot of the compounds in the flavonoid database [1,2]. The Pearson's correlation coefficient r = 0.97

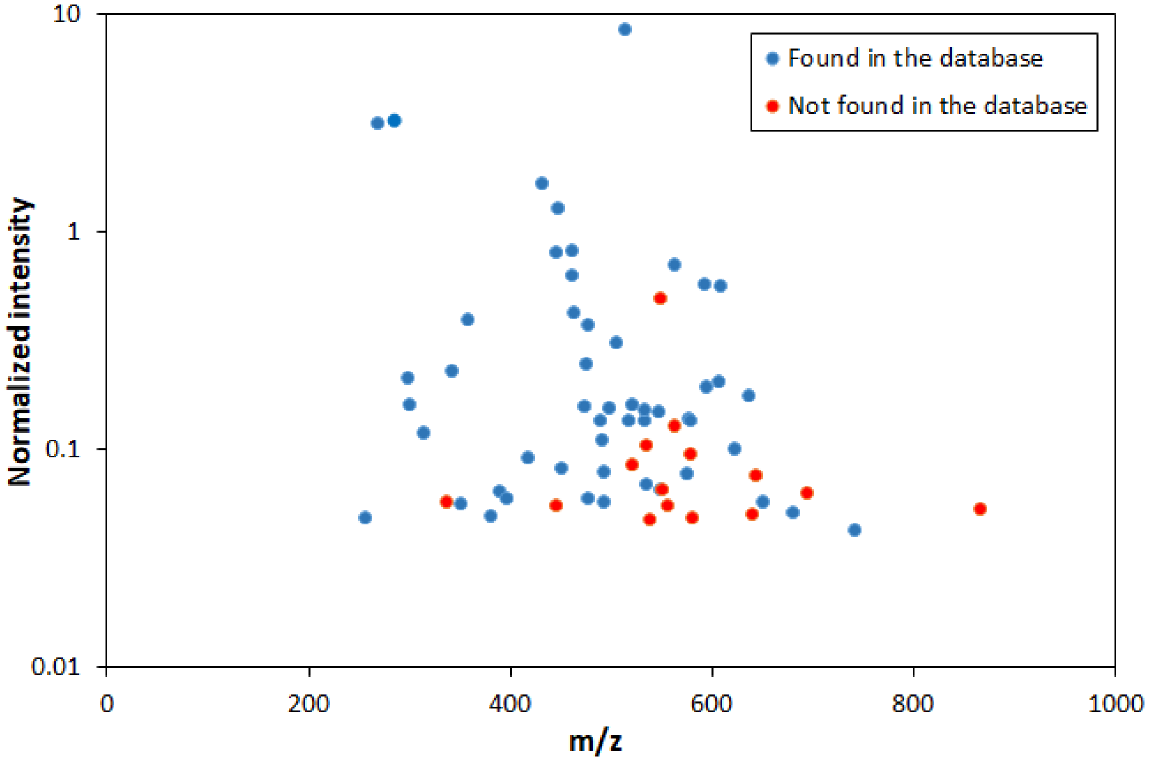

Figure S3. Normalized intensity versus m/z of the mass peaks filtered by M-MARA processing the ESI-DIMS spectra of Sample No. 1 (yarrow extract).

Red dots: suggested as potential flavonoids by M-MARA, but not found in the flavonoid database [1,2].

Characterization of deuterium labeled rutin and quercetin

The Figure S1 shows the UV chromatogram (240 nm) of the synthetized rutin and quercetin. The area fraction of the D-rutin and D-quercetin peaks are higher than 90% measured at 240 nm, that shows the synthetized deuterated compounds have high purity.

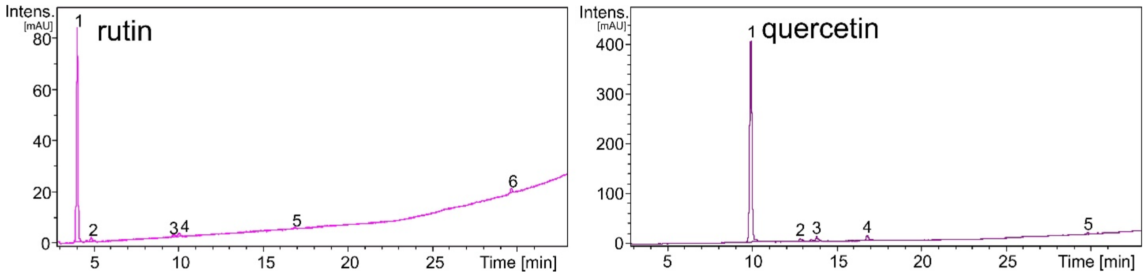

Figure S4. The HPLC-UV chromatograms of th edeuterium labeled rutin and quercetin

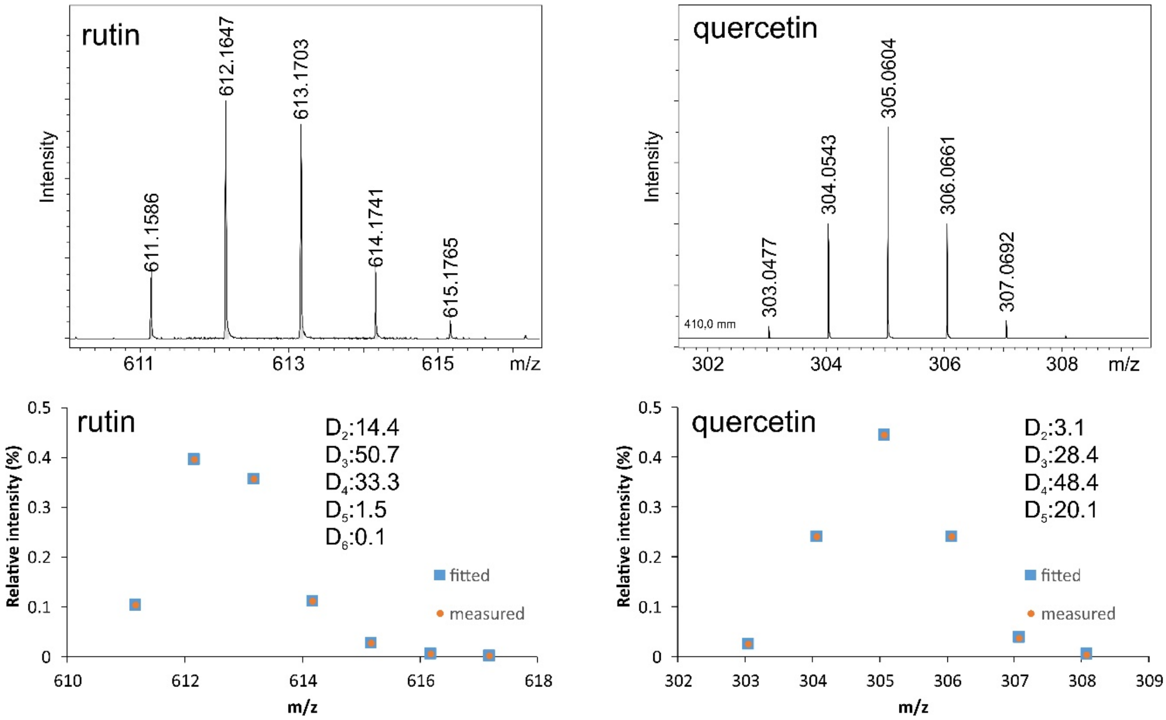

Figure S5. The mass spectra of the deuterium labeled rutin and quercetin and the fitting of the simulated isotope patterns of compounds with different deuterium content.

The deuterium labelling was not 100% in all position, however the prepared product is suitable for the application as internal standard because these isomers are equal in the case of direct injection.

The deuterium labeled rutin was characterized by ESI-(+)MS. The following peaks were identified in the MS spectrum: m/z 611.1586, 612.1647, 613.1703, 614.1741 and 615.1765, the calculated m/z values are 611.1587 and 612.1649 (for other peaks the accuracy is not high because of overlaps), respectively. The identified peaks show that there are rutin molecules with different number of deuterium. The composition was determined based on the fitting of calculated isotopic distribution of all possible

formulas. The results are shown in the SFig X. The quercetin shows similar results than the rutin, there are good agreement among the measured and simulated masses (measured: 304.0543 and 305.0604, simulated : 304.0542 and 305.0605), the deuterium labeling was successful.

References

[1] Flavonoid Database, Arita Laboratory, National Institute of Genetics http://metabolomics.jp/wiki/Category:FL

[2] Arita M., Suwa K. Search Extension Transforms Wiki into a Relational System: a case for flavonoid metabolite database, BioData Mining 1:7, 2008
